# Case Report: *Trichosporon japonicum* Fungemia in a Pediatric Patient With Refractory Acute B Cell Lymphoblastic Leukemia

**DOI:** 10.3389/fped.2022.861476

**Published:** 2022-03-03

**Authors:** Sami Albitar-Nehme, Marilena Agosta, Agata Helena Kowalska, Livia Mancinelli, Manuela Onori, Barbara Lucignano, Giordana Mattana, Francesco Quagliarella, Maria Giuseppina Cefalo, Pietro Merli, Franco Locatelli, Carlo Federico Perno, Paola Bernaschi

**Affiliations:** ^1^Microbiology and Immunology Diagnostics, Istituto di Ricovero e Cura a Carattere Scientifico (IRCCS) Bambino Gesù Children's Hospital, Rome, Italy; ^2^Faculty of Medical Sciences, University College London, London, United Kingdom; ^3^Department of Pediatric Hematology and Oncology and of Cell and Gene Therapy, Istituto di Ricovero e Cura a Carattere Scientifico (IRCCS) Bambino Gesù Children's Hospital, Rome, Italy

**Keywords:** *Trichosporon japonicum*, acute B cell lymphoblastic leukemia, MALDI-TOF MS biotyper, voriconazole, amphotericin B

## Abstract

*Trichosporon japonicum* is a very rare opportunistic yeast causing fungal disease in humans, especially in immunocompromised hosts. Here, we describe a new case of *T. japonicum* isolated from the blood of a pyrexial pediatric patient with refractory acute B cell lymphoblastic leukemia and acute respiratory distress. Prompt diagnosis through early clinical suspicion and appropriate molecular microbiology analysis allowed the yeast to be accurately identified at species level. Subsequent drug susceptibility testing and focused antifungal treatment with voriconazole and amphotericin B led to a complete clinical and mycological resolution of the infection, which represents the second successful case of *T. japonicum* bloodstream infection described in literature to date.

## Introduction

*Trichosporon* species have been recognized as emerging opportunistic yeasts. They are commonly found on the skin and in the gastrointestinal flora of humans in temperate and semitropical climates (1). However, *Trichosporon* spp. can cause invasive and life-threatening fungal disease in immunocompromised hosts (2).

An exceedingly rare representative of this genus, *Trichosporon japonicum*, was first isolated in 1971 from the air of a microbiological laboratory in Japan and named by Sugita and Nakase in 1998 (3). The first clinical case was published in 2008, describing a *T*. *japonicum* related fungemia in a child with acute myeloid leukemia (AML) (4). In this report, we present a case of *T*. *japonicum* infection in a pediatric patient with refractory acute B cell lymphoblastic leukemia. Due to the rarity and growing importance of *T*. *japonicum*, here, we discuss a case which represents the second complete clinical and mycological resolution of *T*. *japonicum* blood infection amongst all case reports in literature to date (5).

## Case Description

### Patient History

An 8-year-old boy was admitted to Bambino Gesù Children's Hospital in October 2020 with a diagnosis of refractory acute B cell lymphoblastic leukemia (ALL) for chimeric antigen receptor (CAR) T-cell therapy in preparation for hematopoietic stem cell transplantation. The diagnosis of ALL had been made in March 2019 in the country of origin of the patient. Subsequently, he had been started on treatment with the first-line chemotherapy protocol (Total XV protocol) which was interrupted due to disease progression. Thereafter, two cycles of Blinatumomab had been tried, however, the second one had been interrupted because of another relapse. Following that, the patient had been given hyperfractionated cyclophosphamide, vincristine sulfate, doxorubicin hydrochloride, and dexamethasone (hyper-CVAD) plus inotuzumab ozogamicin. Finally, he had been transferred to our hospital in Rome.

During his stay in our center treatment was initiated with bridging chemotherapy and bortezomib, followed by two CAR T-cells infusions with poor response. Bone marrow aspirate performed as per protocol 4 weeks after the second infusion of CAR T-cells revealed 90% of CD19-negative blasts. Thus, a therapeutic failure was declared at the end of the fourth month of hospital stay.

### Diagnostic Assessment and Therapeutic Intervention

**Figure 1:**
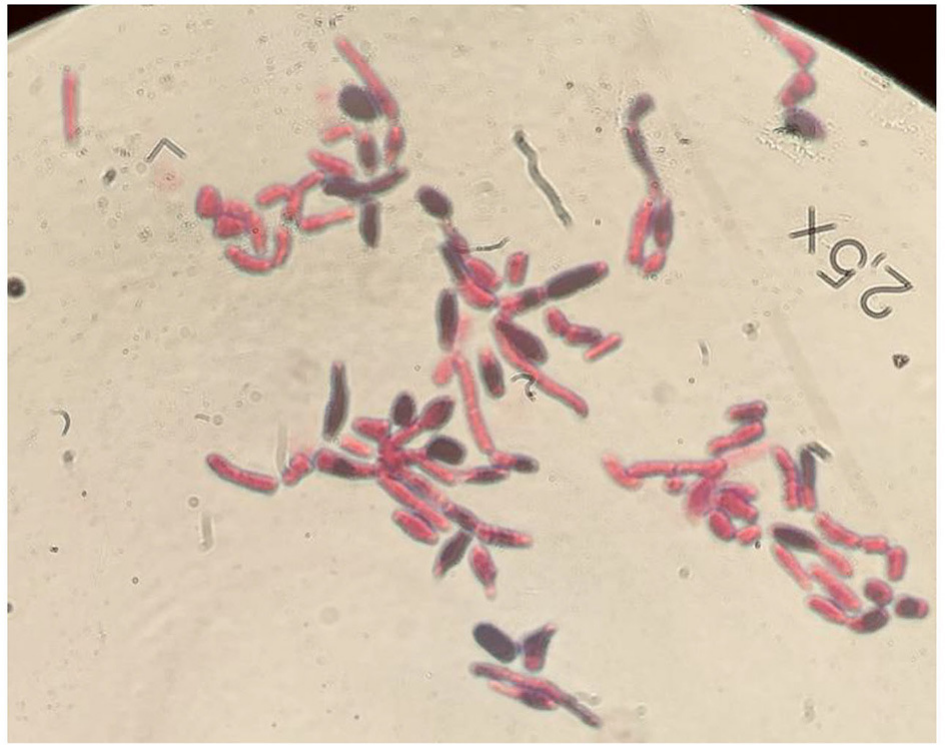
Microscopic appearance of *T*. *japonicum* isolates with Gram stain using RAL-stainer, magnified 100 times.

In the fifth month of admission, the patient, who was on amphotericin B (AMB) long-term prophylaxis (3 mg/kg intravenously once a day), developed fever, acute respiratory distress and cutaneous lesions suggestive of septic emboli on day 1 (Table 1). High level of C-reactive protein (CRP) was measured at 34 mg/dL. In parallel, blood culture (BC) samples were taken from central venous catheter (CVC) for identification of a potential bacterial infection, as well as for fungi detection; all were processed on BD BACTEC™ FX. No *Candida* species were detected on the T2Dx Instrument (T2 Biosystems). Of note, caspofungin (50 mg/m^2^ intravenously once a day) was added to AMB on the same day. On day 2, a whole body computed tomography (CT) showed multiple bilateral lung nodules and micronodules, mainly in centrilobular distribution with confluence in some areas. Evaluation of CT raised suspicion of mycobacterial infection and led to the analysis of expectorated sputum samples and gastric aspirates, which all resulted negative. There was no evidence of central nervous system (CNS) involvement. BCs were repeated on days 2 and 3; together with the BC taken on day 1, they all became positive for fungi on days 4 and 5, respectively. The first hypothetical identification was done by phenotypical microscopic observation (Figure 1). The isolate was then identified as *T*. *japonicum* by matrix-assisted laser desorption/ionization-time of flight mass spectrometry (MALDI-TOF MS, Bruker Biotyper) with a high score value of 2.05. It was further confirmed by molecular typing, using MicroSEQ™ 500 16S rDNA PCR Kit (ThermoFisher SCIENTIFIC) and repeatedly detected on days 4 and 5. Antifungal susceptibility testing was performed according to Clinical and Laboratory Standards Institute (CLSI) guidelines. The isolate of *T*. *japonicum* displayed presumed variable susceptibility as evidenced by the following minimum inhibitory concentrations (MIC): anidulafungin (>8 μg/mL), caspofungin (8 μg/mL) and micafungin (>8 μg/mL); AMB (0.25 μg/mL); fluconazole (1 μg/mL), itraconazole (0.12 μg/mL), posaconazole (0.12 μg/mL), isavuconazole (0.06 μg/mL), and voriconazole (0.03 μg/mL). Based on the antimycogram, caspofungin was changed to voriconazole (8 mg/kg intravenously twice a day) on day 5, while AMB was continued at the same dose. It is important to note that the patient remained neutropenic (neutrophils <100/μL) during all the hospital stay, including the episode of fungemia, and did not recover the neutrophil count upon treatment.

**Table 1:** Timeline of *Trichosporon japonicum* fungemia course.

**Timeline**	**Clinical features and investigations**	**Treatment**
Four months of hospital stay	•Progression of malignancy •Bone marrow aspirate 90% of CD19-negative blast	Bridging chemotherapy and bortezomib, two CAR T-cells therapy infusions, AMB prophylaxis
Day 1 (middle of fifth month of hospital stay)	•Fever, acute respiratory distress and cutaneous lesions •CRP 34 mg/dL	Caspofungin and AMB
Day 2	•Multiple bilateral lung nodules and micronodules on CT	Caspofungin and AMB
Days 4–5	•Blood culture positive for fungi •Identification of *Trichosporon japonicum* on MALDI-TOF •Confirmation of the pathogen by repeated tests including molecular typing	Voriconazole and AMB
Days 9–15	•Negative blood cultures •Rapid and continuous clinical improvement	Voriconazole and AMB
Day 16	•Discharge	Palliative care and antifungal prophylaxis with oral voriconazole

*AMB, amphotericin B; CAR, chimeric antigen receptor; CRP, C-reactive protein; CT, computed tomography; MALDI-TOF, matrix-assisted laser desorption/ionization-time of flight*.

We analyzed all samples in which this yeast was isolated from BCs between days 1 to 15. There was a rapid clinical improvement with a complete resolution of symptoms and the infection was cleared by day 14 as confirmed by repeatedly negative BCs. Nevertheless, due to progression of ALL, it was decided that further treatment should focus only on palliative care. The patient was discharged on antifungal prophylaxis (voriconazole 150 mg by mouth twice a day) and came back to his country of origin where he died 3 weeks later due to the underlying malignancy.

## Discussion

The genus *Trichosporon* is composed of ~50 species which are widely present in nature (soil, air, seawater); 16 species have been identified as human pathogens of which *T*. *japonicum* has been isolated from different sources such as biopsy, skin, sputum, vaginal mucosa, bile, urine, pleural fluid, nails (6, 7). To date, only two clinical cases of *T*. *japonicum* isolated from blood samples have been described in the literature (5, 8). One case was reported in an adult with hematological malignancy who had undergone hematopoietic stem cell transplantation. The infection was catheter-related, which was later removed, and it contributed to death; treatment with liposomal AMB was not adequate (8). Additionally, the very first clinical case concerned a child with AML post bone marrow transplantation. Multiple yeasts were isolated from sputum cultures, therefore, she was treated with liposomal AMB and itraconazole. *T*. *japonicum* was only identified after her death (4). These cases illustrate the difficulty and delay in accurate diagnosis of *T*. *japonicum* infection along with the associated high mortality.

**Figure 2:**
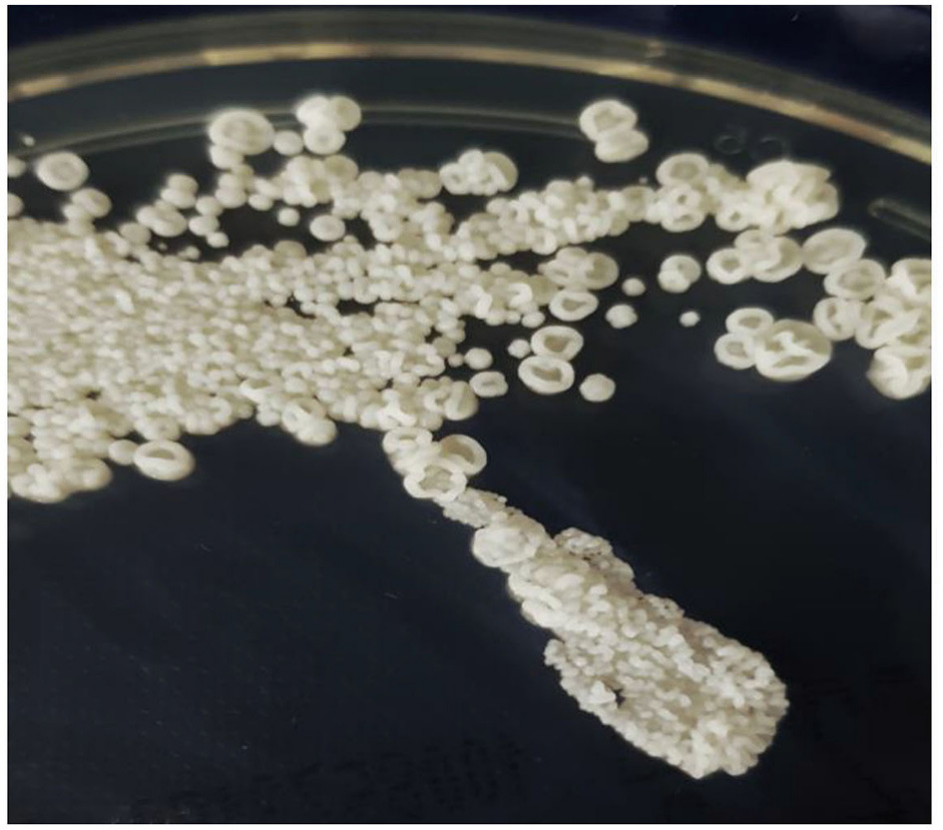
*Trichosporon japonicum* colony morphology, grown on Sabouraud-gentamicin-chloramphenicol-2-agar (SGC2) at 30°C for 4 days (bioMérieux).

Little is known about factors related to *T*. *japonicum* virulence. Thermotolerance is a key feature of pathogenicity in yeasts as it allows them to switch from commensal to a pathogenic lifestyle (2, 7). Our results demonstrated that isolates of *T*. *japonicum* grow at 30°C as well as 37°C (Figure 2). Another known virulence factor of *Trichosporon* spp. is biofilm formation; it has been studied lately in relation to antifungal susceptibility (9). In this regard, Agirbasli et al. described a case of significant biofilm formation in *T*. *japonicum* (4).

Invasive *Trichosporon* infections often cause breakthrough fungemia with high mortality rate particularly in patients with hematological malignancies. These bloodstream infections are primarily associated with the presence of a CVC (10). However, it is somehow tricky to determine the source or portal of entry of the yeast in our case since the symptoms resolved and fungal BCs were repeatedly negative while the CVC was preserved until the end of the patient's hospital stay. In addition, no data suggested that the country of origin could be the reservoir of *T*. *japonicum*.

There is an increased resistance of *Trichosporon* spp. to antifungals as commonly reported in literature (11). Data obtained by Francisco et al. revealed that *Trichosporon* genus exhibits high resistance to AMB which seems to be species specific (12). In our case, the isolates of *T*. *japonicum* showed high MIC for echinocandins and fluconazole, medium for AMB and multiple azoles while the most effective drugs were isavuconazole and voriconazole. The infection occurred despite the use of AMB prophylaxis; however, it was successfully treated with voriconazole given in combination with AMB. Even though the most recent global guidelines give only marginal recommendation for adding AMB to voriconazole (13), the clinical decision to use such combination treatment was supported by the profound aplasia and impracticability of CVC removal.

In this study, despite the conservation of the CVC and the underlying disease progression, a clearance of the fungal infection was obtained with the combination AMB-voriconazole regimen. We therefore demonstrate the important role of voriconazole as a valuable therapeutic tool against *Trichosporon* infections. In conclusion, regardless of the disease stage and presence or absence of clinical manifestations, we recommend a regular focused investigation for fungi and yeasts in neutropenic patients, expanding it beyond the species and genera subject to most common diagnostic procedures.

## Patient Perspective

The parents of the patient gave their consent to all the treatment and the publication of the case report. Unfortunately, the perspective of the patient and their parents were not explored due to their return to the country of origin and terminal condition of the patient.

## Data Availability Statement

The original contributions presented in the study are included in the article/supplementary material, further inquiries can be directed to the corresponding author.

## Ethics Statement

The studies involving human participants were reviewed and approved by Ethics Committee of the Bambino Gesù Children's Hospital. Written informed consent to participate in this study was provided by the participants' legal guardian/next of kin.

## Author Contributions

SA-N, MA, AK, LM, MO, BL, and GM contributed to study design, data collection, analysis and interpretation, and drafting and writing the manuscript. FQ, MC, and PM supported the study with their role and clinical expertise. FL followed the patient outcome and critically revised the manuscript. CP and PB were responsible for funding acquisition and supervised the project. All authors approved the final version to be submitted for publication.

## Conflict of Interest

The authors declare that the research was conducted in the absence of any commercial or financial relationships that could be construed as a potential conflict of interest. The reviewer RH declared a past co-authorship with the authors FL and PM and the absence of any ongoing collaboration with any of the authors to the handling editor.

## Publisher's Note

All claims expressed in this article are solely those of the authors and do not necessarily represent those of their affiliated organizations, or those of the publisher, the editors and the reviewers. Any product that may be evaluated in this article, or claim that may be made by its manufacturer, is not guaranteed or endorsed by the publisher.
